# Artificial Warthog Burrows Used to Sample Adult and Immature Tsetse (*Glossina* spp) in the Zambezi Valley of Zimbabwe

**DOI:** 10.1371/journal.pntd.0003565

**Published:** 2015-03-18

**Authors:** John W. Hargrove, M. Odwell Muzari

**Affiliations:** 1 Centre of Excellence in Epidemiological Modelling and Analysis (SACEMA), University of Stellenbosch, Stellenbosch, South Africa; 2 Public Health Unit, Queensland Health, Cairns, Australia; International Centre of Insect Physiology and Ecology, KENYA

## Abstract

**Background:**

The biology of adult tsetse (*Glossina* spp), vectors of trypanosomiasis in Africa, has been extensively studied – but little is known about larviposition in the field.

**Methodology/Principal Findings:**

In September-November 1998, in the hot-dry season in Zimbabwe’s Zambezi Valley, we used artificial warthog burrows to capture adult females as they deposited larvae. Females were subjected to ovarian dissection and were defined as perinatal flies, assumed to have entered burrows to larviposit, if oocyte sizes indicated >95% pregnancy completion. Perinatal flies were defined as full-term pregnant if there was a late third instar larva *in utero*, or postpartum if the uterus was empty. All other females were defined as pre-full-term pregnant (pre-FT). Of 845 G. m. morsitans captured, 91% (765) were female and 295/724 (41%) of females dissected were perinatal flies. By contrast, of 2805 *G*. *pallidipes* captured only 71% (2003) were female and only 33% (596/1825) of females were perinatal. Among all perinatal females 67% (596/891) were *G*. *pallidipes*. Conversely, in burrows not fitted with traps – such that flies were free to come and go – 1834 (59%) of pupae deposited were *G*. *m*. *morsitans* and only 1297 (41%) were *G*. *pallidipes*. Thus, while more full-term pregnant *G*. *pallidipes* enter burrows, greater proportions of *G*. *m*. *morsitans* larviposit in them, reflecting a greater discrimination among *G*. *pallidipes* in choosing larviposition sites. Catches of males and pre-FT females increased strongly with temperatures above 32°C, indicating that these flies used burrows as refuges from high ambient temperatures. Conversely, catches of perinatal females changed little with maximum temperature but declined from late September through November: females may anticipate that burrows will be inundated during the forthcoming wet season. Ovarian age distributions of perinatal and pre-FT females were similar, consistent with all ages of females larvipositing in burrows with similar probability.

**Conclusions/Significance:**

Artificial warthog burrows provide a novel method for collecting tsetse pupae, studying tsetse behaviour at larviposition, assessing the physiological status of female tsetse and their larvae, and of improving understanding of the physiological dynamics of terminal pregnancy, and population dynamics generally, with a view to improving methods of trypanosomiasis control.

## Author Summary

Adult tsetse, vectors of trypanosomiasis, have been extensively studied for more than 100 years, but little is known about larviposition behaviour in the field. Pupae are generally collected in the field via arduous searches of putative larviposition sites. Females have never been sampled in the field as they deposit a larva, leading to confusion about the physiological dynamics at the end of pregnancy. We overcome these problems through the use of artificial warthog burrows, where tsetse deposit pupae during the hot dry season in the Zambezi Valley of Zimbabwe. When burrows were fitted with a retaining trap it was also possible to sample perinatal (full-term pregnant and postpartum) female tsetse. Comparisons of the numbers of pupae deposited in burrows without the trap, with the numbers of perinatal flies trapped in burrows, showed that many full-term pregnant female tsetse enter burrows but then leave without depositing a larva. *G*. *pallidipes* are more discriminating in this regard than *G*. *m*. *morsitans*. Capture of perinatal females will make it possible, for the first time, to compare the physiological status of female tsetse and the pupa they have just deposited, with important implications for the understanding of tsetse population dynamics.

## Introduction

Adult tsetse flies (*Glossina* spp, Diptera: Glossinidae) are the vectors of human and animal trypanosomiasis in Africa and, as such, have been the object of intense study since the early 20^th^ century and the source of an extensive literature on their field biology. Our knowledge of the (briefly free-living) larval and pupal stages is, by contrast, limited mainly to laboratory studies [1,2,3,4,5,6,7]. Studies on the physiology of reproduction in tsetse[8], showed that they have a very unusual reproductive system, termed *adenotrophic viviparity*. Unlike most Diptera, which lay large numbers of small eggs that hatch to produce free-living larvae, tsetse have just two ovarioles in each of the left and right ovaries and only produce one large egg at a time. The egg is retained in the uterus, fertilized there by sperm stored in the female’s spermathecae, and hatches after 4–6 days to produce a first instar larva. This larval stage, and two subsequent instars, are fed via a highly modified uterine or milk gland producing ultimately a late third instar larva that often constitutes more than 50% of the female’s total body mass. In the field a full-term third instar larva is typically deposited in sand, or soft soil, covered with leaf litter. The larva burrows a few centimetres into the substrate and rapidly forms around itself a hard, waterproof, puparial case of chitin. Inside this puparial case the fly goes through the larval, pre-pupal, pupal and pharate adult stages before emerging as the teneral adult fly some weeks after larviposition, the period depending on temperature.

Whereas this process has been studied in the laboratory, it is extremely unusual to observe female tsetse larvipositing in the field: indeed it is uncommon to find female tsetse with a full-term larva *in utero* and this difficulty has led to some uncertainty regarding the physiological status of female flies at terminal stages of pregnancy [9]. Field studies have accordingly been limited to finding puparia that have already been deposited. This involves methodical searches of suspected larviposition sites—under fallen logs and rocks, in rot-holes in trees, in shaded places on the edges of dry river-beds, or in loose soil, or sand, covered with leaf litter [10,11].

In Zimbabwe, from August to November, tsetse puparia can frequently be found in burrows dug by the aardvark *Orycteropus afer* Pallas and often used thereafter by warthog *Phacochoerus aethiopicus* Pallas [11,12]. Muzari & Hargrove showed that female tsetse larviposit in artificial versions of these ‘warthog burrows’[13]. They described the construction of such devices and recorded annual variations in their use as larviposition sites by the tsetse flies *Glossina morsitans morsitans* Westwood and *G*. *pallidipes* Austen at Rekomitjie Research Station in the Zambezi Valley of Zimbabwe. The original study was concerned only with the collection of puparia, but a device was also described whereby flies could be captured in the burrows. The primary purpose of the present study was, for the first time in tsetse fieldwork, to sample full-term pregnant flies just before they deposited a larva. More generally we studied the sample characteristics of this new capture system. Our null hypotheses were that: the exact location of larviposition was independent of burrow site and orientation; that the larviposition site within each burrow was randomly chosen; that these choices were the same for both species and for females of different ages; that the timing of larviposition was random throughout the day and that a full-term pregnant fly entering a burrow deposited its larva in that burrow.

Since warthog burrows are always cooler than ambient during daylight hours [13], it seemed likely that tsetse would also use the burrows as “refuge” sites—the cooler dark places where flies are found in increasingly large numbers as temperatures increase above 32^°^C [12,14]. Accordingly we also studied the effects of calendar time and temperature on the sex and species distribution of captured flies and, particularly for female flies, the distribution between full-term pregnant, and other, females.

The study suggested that female tsetse do not larviposit randomly with respect to time, nor with position within burrows, and that full-term flies often enter a burrow but then leave it without larvipositing. This behaviour differed between species. The research opens the door for an entirely new area of tsetse studies—including comparisons between the physiological status of individual females and the pupae they have just deposited.

## Materials and Methods

### Artificial warthog burrows

**Fig 1:**
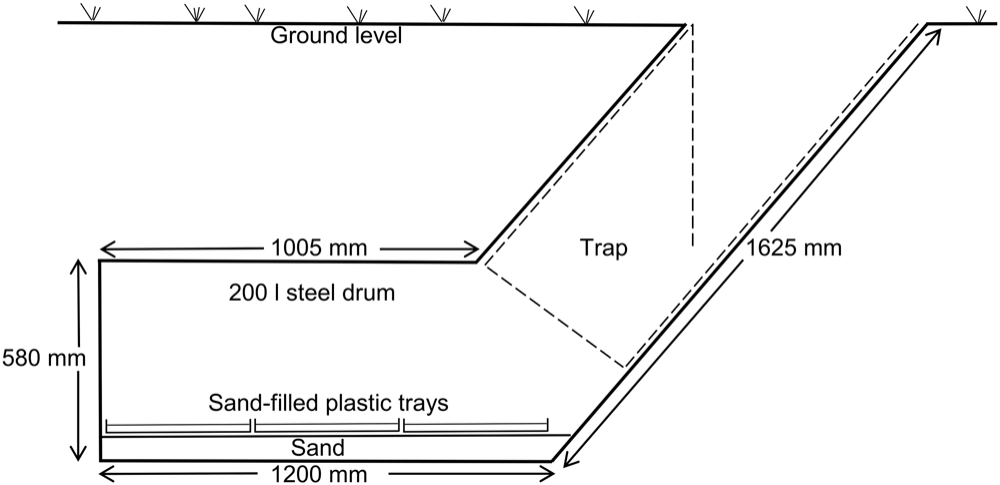
Section through an artificial warthog burrow. Dashed lines indicate the frame of the box-shaped trap that could be inserted into the entrance of the burrow. The trap frame was made from 8-gauge wire and was covered with black netting of fine cloth fabric. The trap fills the mouth of the burrow, but has an opening allowing entry of flies into the trap. Flies entering the burrow flew under the lip of the trap and were retained when they headed towards the light. When the trap was not in place, female flies could deposit their larvae in the plastic trays, which were half-filled with sand, on top of which was placed a *c*. 2cm layer of leaf litter.

During 1998, 36 artificial warthog burrows were deployed over a distance of 800m along the banks of the Chiuyi and Rukomechi Rivers near Rekomitjie Research Station, Zambezi Valley, Zimbabwe. Burrows were deployed at nine sites in groups of four, with burrow openings at each site facing north, east, south and west, respectively. Burrow construction (Fig. 1) is fully described by Muzari & Hargrove (2005).

For each of the 20 burrows at sites 1 to 5 a wire-framed, net-covered, trap was inserted into the mouth of a burrow, such that tsetse could enter the burrow but were trapped when they flew out towards the light (Fig. 1). Between 8 September and 25 November traps were cleared daily at *c*. 1115, 1230, 1400 and 1645 h. With this regularity of clearing, it was possible to collect “perinatal” female flies—*i*.*e*., postpartum flies that had just deposited a larva, or full-term pregnancy flies which generally produced a larva soon after capture. Male flies, and females that were not perinatal, were also collected and counted.

The 16 burrows at sites 6 to 9 were always used without a trap so that adult females could enter and leave the burrow unhindered. Flies entering these burrows could deposit larvae in one of six sand-filled plastic trays placed on the bottom of the burrow or could leave the burrow without depositing a pupa. The positions of any puparia deposited were noted as being found in the left or right column of trays and in the back, middle or front tray, as viewed from the burrow entrance. The idea of this experiment was to study larviposition site selection, in terms of burrow orientation and position within the burrow, with a view to maximising future puparial collection rates. The experiment also facilitated study of the variation of larviposition rates, for each of the two tsetse species, as a function of calendar date and temperature. Puparia were collected from the trays in these burrows, at 3–18 day intervals, between 28 August and 8 November.

### Ovarian dissection

Adult tsetse from burrow traps were placed, with any puparium found in the trap that could be attributed unequivocally to the female fly in question, in individually labelled (75 x 25)-mm glass tubes which were kept under a black cloth in a polystyrene box. Larvae could be attributed unequivocally to their mothers if there was only a single larva/pupa, and only one postpartum female, in the trap cage—or if a full-term pregnant female was transferred from the capture cage to a glass tube and the female thereafter deposited a larva prior to dissection.

Adult females were subjected to ovarian dissection, 98% on their day of capture and the remainder the following morning, and assigned to one of eight ovarian age categories, using the disposition and relative sizes of the oocytes within the ovarioles in the left and right ovaries [15]. This procedure can be used to determine unequivocally the number of times a fly has ovulated as long as this number is less than four. Thereafter, the ovarian category can only be defined modulo 4 [15]: thus, it is not possible to differentiate flies that have ovulated four times from those that have ovulated 8, 12, 16 *etc*. times, and similar problems exist for those that have ovulated 5, 6 or 7 times. This did not constitute a problem for the present study since we were not concerned with actual ages of flies but only the ovarian age distributions of different groups.

Pregnancy stage was assessed from the linear dimensions of the ovarian and uterine contents [16,17]. Females which were seen to have produced a larva after the time of capture—either in the trap or in the collection tube after removal from the trap, but before ovarian dissection, were classified as “postpartum”. This category was also used for flies which had an empty uterus, and where the sizes of the first and second largest oocytes indicated that the female had completed >95% of the ovulation cycle. It was assumed that these flies had very recently larviposited. Females with a third instar larva *in utero*, of a size indicating that >95% of the pregnancy was complete, were classified as “full-term” pregnant. Postpartum and full-term flies were jointly referred to as “perinatal”. All other female flies were termed “pre-full-term” (abbreviated below to pre-FT). This group includes flies in ovarian category zero, *i*.*e*., flies which had not yet ovulated for the first time, and all flies with either an egg or a first, second, or small third instar larva *in utero*. Finally it includes flies with an empty uterus where the size of the oocytes in the ovaries indicated that the female had completed ≤95% of the ovulation cycle. Note that the empty uterus could have occurred due to natural causes, or to capture-related trauma. For present purposes the cause is not relevant: if oocyte size indicates that ≤95% of pregnancy has been completed then the fly is properly classified as pre-FT—regardless of the content of the uterus or, where the uterus is empty, how this came about.

### Meteorological measurements

A mercury thermometer in a Stevenson screen at Rekomitjie Research Station was used to record daily maximum and minimum temperatures. A rain gauge sited next to the screen produced daily records of precipitation. Hourly mean measurements of shade temperature and relative humidity were also made using an automatic weather station (type WS01, Delta-T devices, Newmarket, UK) at a site *c*. 200 m from the Stevenson screen.

### Statistical procedures

Analyses were carried out using Stata (StataCorp, 1999) statistical package, version 12. We denote a chi-squared statistic with n degrees of freedom as χ^2^(n): we used Yates correction whenever n = 1. We denote statistical significance at the 0.05, 0.01 and 0.001 levels of probability by *, ** and ***, respectively: *P*>0.05 is denoted by “ns”. All error terms are 95% confidence intervals (95% ci).

## Results

### Meteorological conditions during the experiment

**Fig 2:**
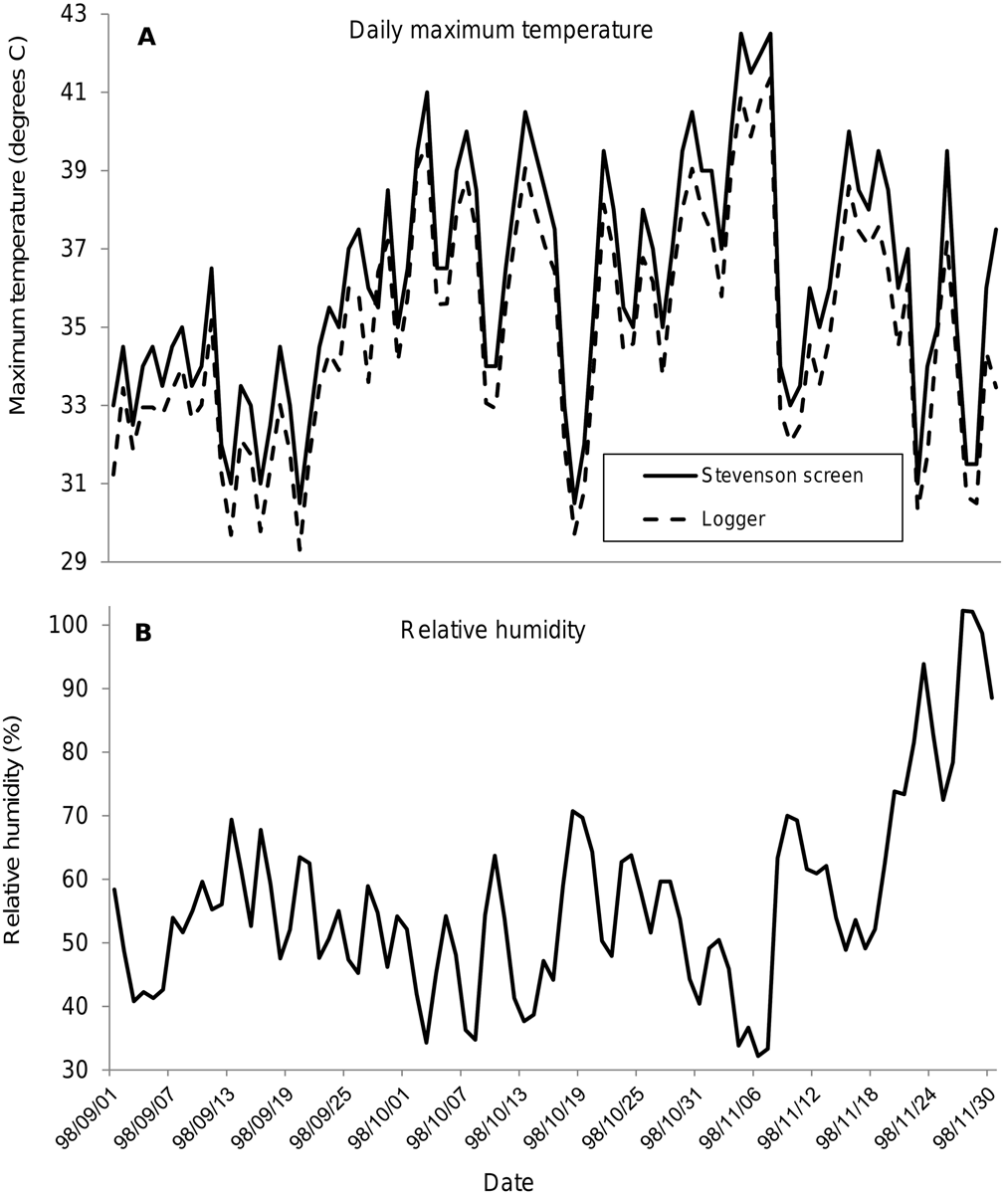
Meteorological conditions at Rekomitjie. Daily maximum temperatures (A) and mean daily relative humidity (B) recorded from an automatic meteorological station at Rekomitjie Research Station, September—November 1998. Maximum temperatures were also recorded from a mercury thermometer in a Stevenson screen.

September marks the onset of the hot-dry season in the Zambezi Valley and temperatures typically increase steadily through October and into November, remaining high until the onset of the rains, which do not generally start in earnest until mid-December. In 1998 maximum temperatures (*Tmax*) increased steadily into mid-October, fell for a few days, then increased again until early November (Fig. 2A), peaking at 42.5^°^C—at that time the highest temperature ever recorded at Rekomitjie. Readings of *Tmax* from the mercury thermometer were on average about 1^°^C higher than those from the logger. The transient declines in October temperatures were associated with increased cloud cover and increases in the relative humidity (Fig. 2B). Rain measured at 0.5, 1.5, and 17mm fell on 19, 20 and 23 November: heavier and more sustained rain fell in December.

Ambient temperatures at the weather station showed strong diurnal changes, with a minimum at about dawn and a maximum at 1500–1600 h. Despite the high ambient temperatures and the large diurnal variation, mean temperatures varied only between 28 and 29.5^°^C in the well-insulated burrows, which were up to 3^°^C cooler even than artificial refuges during daylight hours—but were warmer between 2200 h and shortly after dawn [13].

### Rates of larviposition in artificial warthog burrows

Of 3131 tsetse puparia collected from sand-filled trays in the 16 burrows at sites 6 to 9, 1834 (59%; 95% ci 57%- 60%) were *G*. *m*. *morsitans* and 1297 (41%; 95% ci 40%- 43%) were *G*. *pallidipes* (Table 1, 2). There was no significant difference between the proportions of puparia found in the trays on the left or right side of the burrow (χ^2^(1) = 0.2, *P* > 0.05: Table 1) but the vast majority of both species deposited their larvae in the trays at the back of (deepest into) the burrow (χ^2^(2) >1300, P< 0.001 for each species: Table 1), and the smallest proportion in the trays closest to the mouth. There was also a significant difference between species in this regard, with *G*. *pallidipes* shifted significantly more towards the trays at the back of the burrow than *G*. *m*. *morsitans* (χ^2^(2) = 48.1, P< 0.001).

**Table 1:** The distribution of tsetse puparia collected from 16 artificial warthog burrows (sites 6 to 9) at Rekomitjie Research Station, 28 August – 8 November 1998.

	*G*. *m*. *morsitans*				*G*. *pallidipes*			
	Left	Right	Total	%	Left	Right	Total	%
Back	675	676	1351	73.7	550	528	1078	83.1
Middle	212	159	371	20.2	99	92	191	14.7
Front	45	67	112	6.1	11	17	28	2.2
		Total	1834			Total	1297	
Puparia per site per day			1.74				1.23	

Distribution between six trays placed in each burrow. Percentages refer to distribution between back, middle and front trays.

**Table 2:** The distribution of tsetse puparia collected from 16 artificial warthog burrows (sites 6 to 9) at Rekomitjie Research Station, 28 August – 8 November 1998.

Site	*G*. *m*. *morsitans*	%	*G*. *pallidipes*	%	Total
6	119	55.1	97	44.9	216
7	788	57.2	589	42.8	1377
8	491	51.1	470	48.9	961
9	436	75.6	141	24.4	577
Totals	1834	58.6	1297	41.4	3131

Distribution by site and species. Percentages refer to distribution between species.

Other analyses showed that there were significant differences between the proportions of puparia found in burrows facing north, south, east or west (χ^2^(3) = 18.0 and 9.8 for *G*. *m*. *morsitans* and *G*. *pallidipes*, respectively: P<0.001 in each case). There were, nonetheless, fairly small deviations from a uniform distribution of 25% in each burrow: for *G*. *m*. *morsitans* the range was 22.3%- 29.2% and for *G*. *pallidipes* 21.8%- 27.9%. The largest proportions were found in west and north-facing burrows for the two species, respectively. The data in Table 1 are for puparial numbers pooled on all sites, but the distribution of puparia between trays within a given burrow, and between burrows at a given site, were all similar. Accordingly, all further analyses of these data are carried out on data pooled on burrow for each site.

In summary, burrow orientation was of minor importance for larviposition: but for both species, and particularly *G*. *pallidipes*, the vast majority of larvae were deposited at the very rear of the burrow, raising the possibility that deeper/longer burrows might have encouraged greater rates of larviposition.

**Fig 3:**
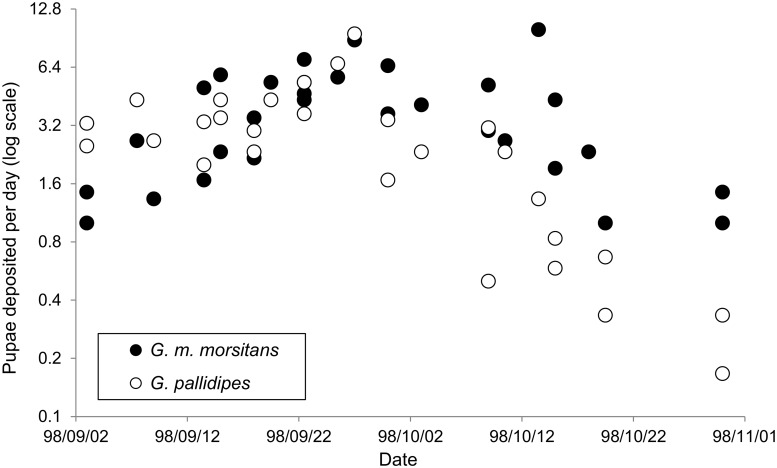
Changing rates of pupal deposition in artificial warthog burrows. Mean numbers of *G*. *m*. *morsitans* and *G*. *pallidipes* puparia collected daily from four burrows at site 7, Rekomitjie Research Station between 25 August and 21 October 1998.

The total number of puparia collected varied between the four sites by a factor of 6.4:1. There were always fewer *G*. *pallidipes* than *G*. *m*. *morsitans* but the proportion of *G*. *pallidipes* was much smaller at site 9 than at the other three sites (Table 2). For both species, the number of puparia collected per site per day also changed with calendar time, with a peak at the end of September (Fig. 3). Numbers then declined steadily until mid-November: thereafter almost no puparia are found in burrows [13]. Multiple linear regression analysis indicated, for both species, linear and quadratic effects of time after 28 August, and significant site effects (Table 3).

**Table 3:** Experiment 1.

	*G*. *m*. *morsitans*; *R* ^2^ = 0.62	*G*. *pallidipes*; *R* ^2^ = 0.49
Site 6	0	0
Site 7	1.02 (0.84–1.20)***	0.83 (0.63–1.04)***
Site 8	0.68 (0.50–0.85)***	0.58 (0.37–0.79)***
Site 9	0.62 (0.44–0.79)***	0.13 (-0.08–0.34)ns
*t*	0.61 (0.46–0.77)***	0.24 (0.06–0.43)***
*t* ^2^	-0.082 (-0.103 –-0.060)***	-0.049 (-0.075 –-0.024)***

The effects of calendar date (t) and burrow siting on the numbers of tsetse puparia collected from trays placed in 16 burrows at sites 6 to 9 at Rekomitjie Research Station, 28 August—8 November 1998. The dependent variable was loge(n+1) where n was the number of puparia collected per day. Site 6 was used as the reference for estimating site effects. R2 for the models were 0.62 and 0.49 for G. m. morsitans and G. pallidipes, respectively.

### Catches of tsetse from artificial warthog burrows

What is not clear from the above results is the extent to which changes in larviposition rates reflect changes in tsetse population levels, as opposed to changes in the probability that females used burrows as larviposition sites. Moreover, it is not clear how day-to-day variation in larviposition rates in burrows is affected by meteorological factors. These issues were investigated in experiments where tsetse were captured in traps inserted into burrow mouths (Fig. 1).

Some care is required in the analysis of these results since both males and females of both species are now captured. Since burrows provide dark spaces where daytime temperatures are always markedly below ambient [13], male flies, as well as females that are not about to deposit a larva, are likely to use burrows not for larviposition but as artificial “refuges” from the heat [12]. For the sampling period 8 September—25 November the total catches of male and female tsetse from the burrows at sites 1 to 5 were 80 and 765 for *G*. *m*. *morsitans*, and 802 and 2003 for *G*. *pallidipes*. Of the females captured, 295/724 (41%; 95% ci 37%- 44%) of *G*. *m*. *morsitans* were successfully dissected and identified as perinatal (Table 4), using the definitions described in the Methods. This was a significantly higher proportion than the 596/1825 (33%; 95% ci 31%- 35%) of *G*. *pallidipes* identified as perinatal.

**Table 4:** Species distribution (percentages in parentheses) of pre-full-term-pregnancy and perinatal female tsetse caught in 20 burrows at sites 1 to 5 at Rekomitjie Research Station, 8 September – 25 November 1998.

Month	Group	*G*. *m*. *morsitans*	*G*. *pallidipes*
Sept	Pre-FT	39 (33.6)	77 (66.4)
	Perinatal	221 (34.8)	415 (65.2)ns
Oct	Pre-FT	257 (25.8)	741 (74.2)
	Perinatal	66 (29.5)	158 (70.5)ns
Nov	Pre-FT	133 (24.5)	411 (75.5)
	Perinatal	8 (25.8)	23 (74.2)ns
Totals	Pre-FT	429 (25.9)	1229 (74.1)
	Perinatal	295 (33.1)	596 (66.9)***
	Total	724	1825

When the data for each month were analysed separately, the proportions of *G*. *m*. *morsitans* and *G*. *pallidipes* among perinatal and pre-FT females did not differ significantly (χ^2^(1) <1.4 in each month, *P*> 0.05: Table 4). When data were pooled over all months, however, there was a significantly greater proportion (33%: 95% ci 30%- 36%) of *G*. *m*. *morsitans* among the perinatal females than among the pre-FT group (26%: 95% ci 24%- 28%) (Table 4).

In contrast to these figures, 1834/3131 (59%: 95% ci 57%- 60%) of the puparia collected from burrow trays were *G*. *m*. *morsitans* (Table 1). For September and October combined, the numbers of perinatal females captured per site per day were 1.65 and 3.29 for *G*. *m*. *morsitans* and *G*. *pallidipes*, respectively, compared with 1.74 and 1.23 puparia of these species collected per site per day (data from Tables 1 and 4).

In short, whereas many more full-term-pregnant *G*. *pallidipes* enter burrows, a greater proportion of *G*. *m*. *morsitans* actually larviposit in those burrows, suggesting that greater proportions of full-term *G*. *pallidipes* leave burrows they have entered without larvipositing.

### Changes with time in the numbers of pre-FT and perinatal females trapped in burrows

**Fig 4:**
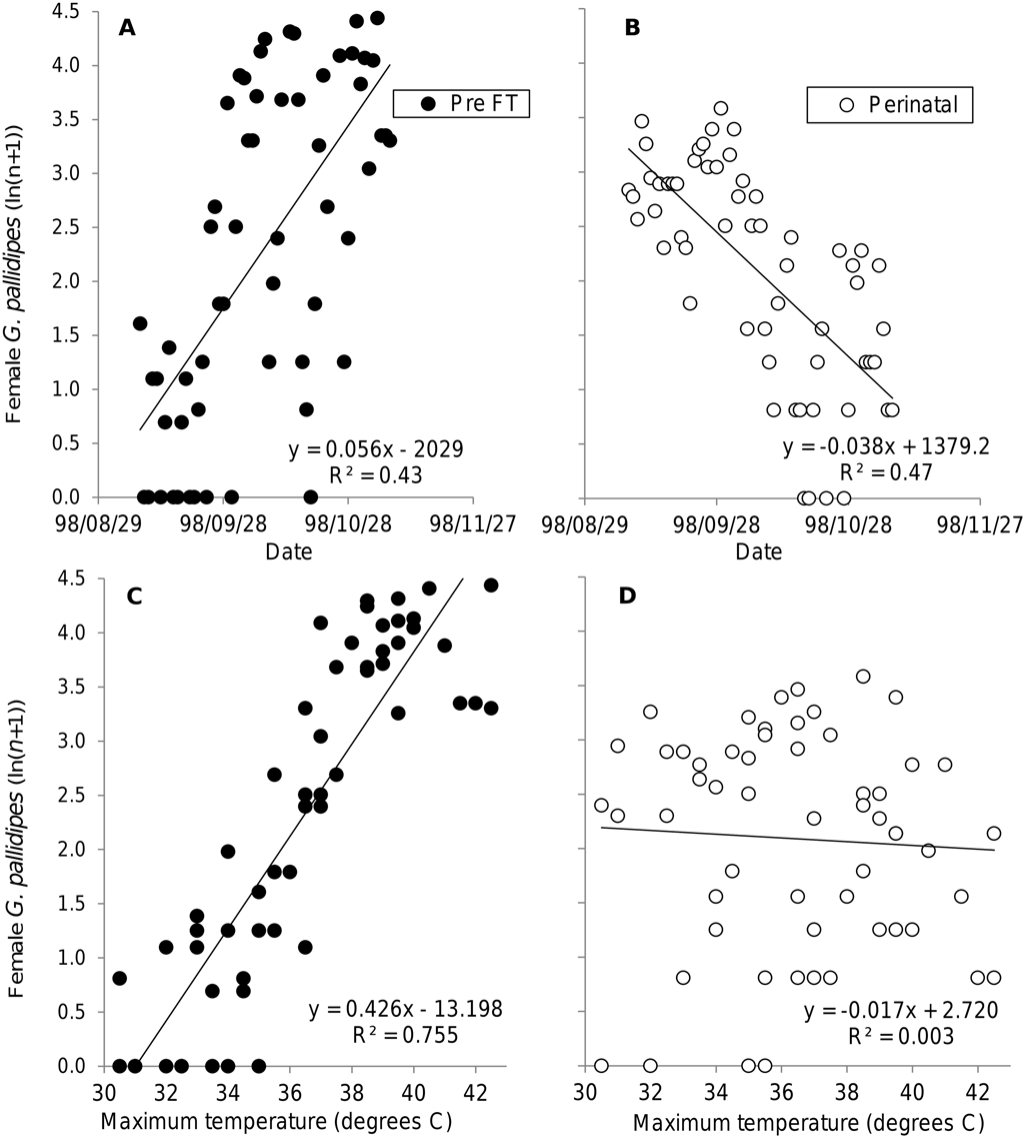
Catches of tsetse from artificial warthog burrows. Changes with time and daily maximum temperature in the numbers of pre-full-term-pregnancy (Pre-FT: A, C) and perinatal (B, D) female *G*. *pallidipes* captured per day in artificial warthog burrows at sites 1 to 5 at Rekomitjie Research Station. All data transformed to log_e_(*n*+1). Data shown for period 8 September to 7 November 1998: thereafter there were many zero catches.

Catches of pre-FT and perinatal female flies showed entirely different trends with calendar time: in univariate analyses, numbers of pre-FT *G*. *pallidipes* increased markedly with time during the experiment (Fig. 4A), whereas exactly the opposite trend was seen among perinatal females of this species (Fig. 4B). Similarly, increasing *Tmax* was associated with rapidly increasing catches of pre-FT flies (Fig. 4C), whereas there was no effect on the numbers of perinatal females captured (Fig. 4D).

Maximum temperature, relative humidity and calendar time are clearly correlated over the period of the experiment and, to tease out their effects on rates of larviposition, multivariate linear regression analysis was carried out on the data for both tsetse species. For pre-FT females of both species, once the very strong temperature effect was removed, a quadratic effect of time was also evident: *i*.*e*., the numbers captured initially increased, and then decreased as the hot weather continued (Table 5). Since temperature is correlated both with relative humidity and saturation deficit, the latter two both had significant effects on catches when used in univariate analyses: the effects were, however, smaller than the temperature effects and, once temperature had been included in the model, there were no detectible additional effects of either relative humidity or saturation deficit on the catches.

**Table 5:** The effects of calendar date (*t* and *t* ^2^) and maximum temperature (*Tmax*) on daily catches of male *G*. *m*. *morsitans* and *G*. *pallidipes*, and females that were either pre-full-term pregnancy (pre-FT) or perinatal, from a trap inserted into the entrances of 20 burrows at sites 1 to 5 at Rekomitjie Research Station, 8 September – 25 November 1998.

*G*. *pallidipes*	Males	Females	Females
	*R* ^2^ = 0.78	Pre-FT: *R* ^2^ = 0.78	Perinatal: *R* ^2^ = 0.60
Constant	-11.3 (-13.2 –-9.32)	-13.4 (-15.6 –-11.1)***	-2.06 (-3.64 –-0.48)*
*t*	6.68 (3.80–9.57)***	6.78 (3.35–10.2)***	-5.01 (-5.60 –-4.42)***
*t* ^2^	-8.94 (-12.4 –-5.45)***	-9.09 (-13.1 –-5.04)***	-
*Tmax*	0.34 (0.28–0.40)***	0.40 (0.34–0.47)***	0.16 (0.11–0.20)***
*G*. *m*. *morsitans*	*R* ^2^ = 0.44	Pre-FT: *R* ^2^ = 0.69	Perinatal: *R* ^2^ = 0.80
Constant	-5.14 (-6.65 –-3.63)	-6.91 (-8.71 –-5.10)***	0.92 (-1.01–2.84)ns
*t*	-	7.93 (5.16–10.70)***	-3.63 (-4.35 –-2.91)***
*t* ^2^	-	-10.6 (-13.9 –-7.33)***	-
*Tmax*	0.16 (0.12–0.20)***	0.21 (0.15–0.26)***	0.047 (-0.010–0.102)ns

The dependent variable was log_e_(*n*+1) where *n* was the number of flies captured per day.

For perinatal females of both species, catches declined significantly with time and increased with temperature—though the latter effect was not significant for *G*. *m*. *morsitans*. The effects of time and temperature on catches of male *G*. *pallidipes* were qualitatively the same as for pre-FT females and the values of the coefficients for the time and temperature effects were not significantly different in the two groups (Table 5). For *G*. *m*. *morsitans* males only 80 flies were caught over the whole experiment and only a positive effect of temperature was found.

### Predicted changes in catches of *G*. *pallidipes* from burrows

**Fig 5:**
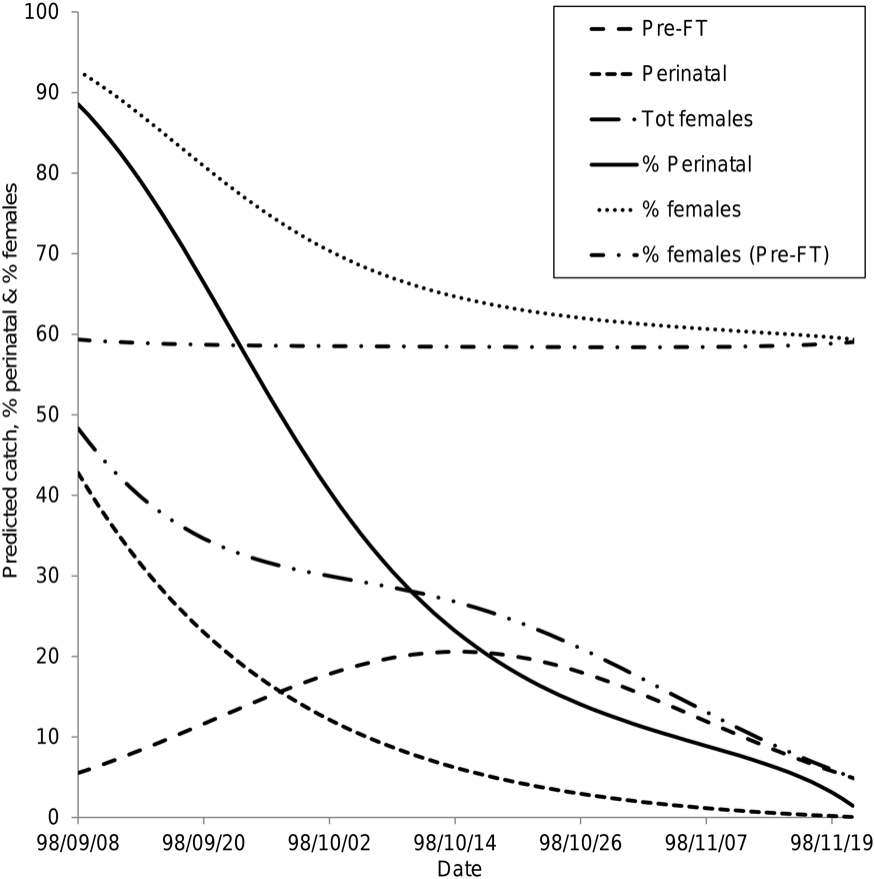
Predicted catches of tsetse from artificial warthog burrows. Catches of male, and of pre-FT and perinatal female, *G*. *pallidipes* from artificial warthog burrows, predicted using the model in Table 5, assuming a constant daily maximum temperature of 37.5°C. The predicted catches were also used to estimate the proportions of perinatal flies among catches of females, and the proportions of females in the whole catch, and among pre-FT flies assumed to be using burrows as a refuge from high temperatures.

When the relationships in Table 5 were used to predict the changes in catches of *G*. *pallidipes*, for given fixed *Tmax*, predicted catches of pre-FT females peaked at the end of the second week in October (Fig. 5). By contrast, the catches of perinatal females decreased from the time that sampling started in early September. There was also a positive effect of *Tmax* on the numbers of perinatal female *G*. *pallidipes* captured, though the coefficient was much smaller than for pre-FT females (Table 5). Accordingly, the proportion of perinatal females in the catch also declined continuously after the beginning of September (Fig. 5).

The model for male *G*. *pallidipes* was closely similar to that for pre-FT females and the predicted catches at a fixed temperature also peaked at the end of the second week of October. Since the numbers of perinatal female flies decreased monotonically with time for given *Tmax*, whereas catches of both males and females reached a peak in mid-October and then declined, the predicted proportion of female flies in the entire catch also declined with time (Fig. 5). If, however, the perinatal females were excluded from the above calculation—so that we were only considering flies assumed to be using burrows as refuges, rather than as larviposition sites, then the predicted percentage of females in the catch was almost constant at about 59% (Fig. 5).

The plots in Fig. 5 are for an arbitrary choice of constant *Tmax*, but choices in the range 35–40°C gave qualitatively similar results: increasing temperature resulted in modest increases in the numbers of perinatal flies, larger increases in the pre-FT group, and thus lower overall percentages of perinatal flies. The percentage of females among flies assumed to be using the burrows as refuges was approximately constant at each temperature, the level shifting from about 55% to 62% as the temperature increased. The models in Table 5 predict that, at constant temperature, 68–80% of catches of pre-FT females will be *G*. *pallidipes*.

### Diurnal distribution of catches

**Fig 6:**
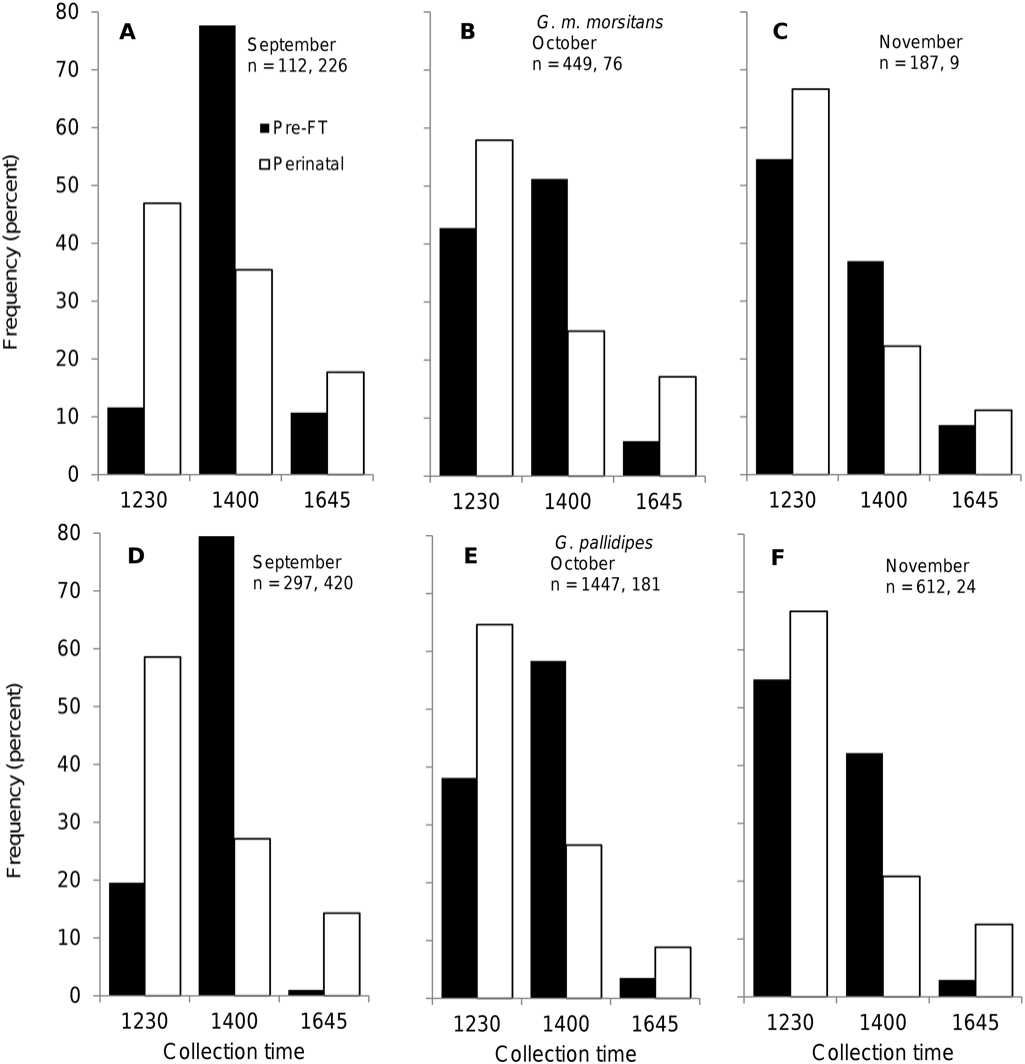
Diurnal variation in catches of female tsetse from artificial warthog burrows. Diurnal distribution of daily catches (*n*) of pre-full-term-pregnancy (Pre-FT) and perinatal *G*. *m*. *morsitans* (A, B, C) and *G*. *pallidipes* (D, E, F) from 20 artificial warthog burrows (sites 1 to 5) deployed at Rekomitjie Research Station, September—November 1998.

The distribution of catches of female flies across the day differed significantly between the two groups of female tsetse (Fig. 6). For the pre-FT group the situation changed with the month of the experiment: in September only about 10% of the *G*. *m*. *morsitans* (Fig. 6A) and 20% of the *G*. *pallidipes* (Fig. 6D) were taken in the 1230h sample. This proportion increased markedly in October, and by November the greatest proportion was caught at this time (Fig. 6C, F). These results are consistent with earlier findings that tsetse enter refuges at increasingly early hours of the day as temperatures increase [12,14]. The perinatal group of flies entered early in the day in all three months: for both species the greatest proportion (40–65%) of the flies were caught in the first sample taken at 1230h, and 80–90% of the days’ samples were caught by 1400h, regardless of the temperature (Fig. 6).

### Age distribution of female tsetse caught in burrows

**Fig 7:**
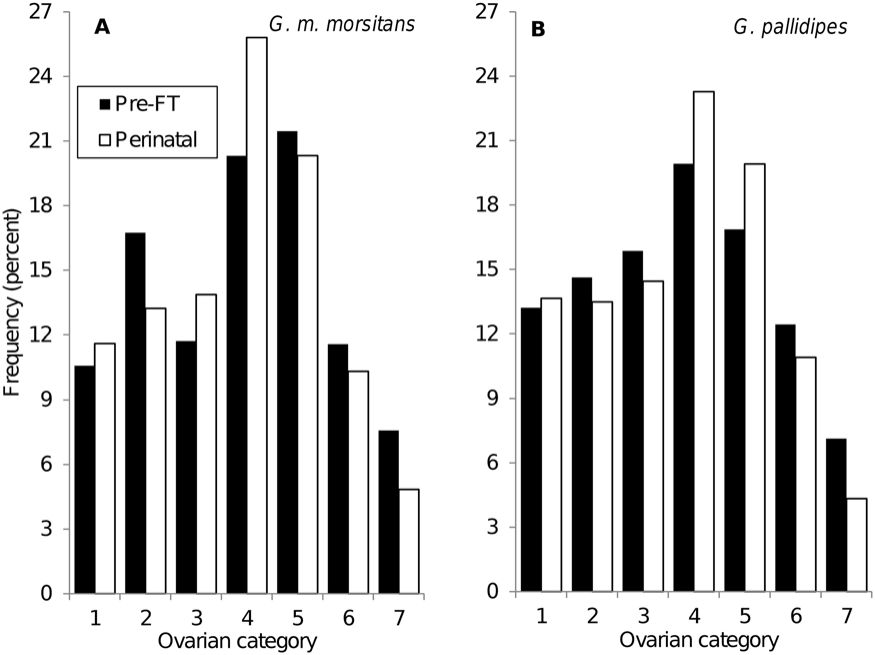
Ages of female tsetse caught in artificial warthog burrows. Ovarian age distributions of female *G*. *m*. *morsitans* (A) and *G*. *pallidipes* (B) captured in all artificial warthog burrows September—November 1998. Ovarian category zero flies, which cannot be in the perinatal group, have been omitted. Postpartum flies were assigned to the ovarian stage that they had just completed.

An obvious difference between the age structure of the pre-FT and perinatal groups of female is that none of the latter can be flies in ovarian category zero, which have not yet ovulated for the first time. When this age category was excluded from the analysis there was little difference, for either species, between the age structures of the two groups (Fig. 7).

## Discussion

We describe for the first time the characteristics of two species of tsetse caught using a new sampling system, artificial warthog burrows. The burrows were initially designed to provide a simple method for collecting tsetse puparia in the field. It became apparent, however, that the additional use of a trap allowed us to sample adult flies entering the burrows. Initial trials of this system in August 1997 showed that, at that time of the year, the catch consisted entirely of female tsetse that all deposited a larva in the trap. When, as in the current study, sampling was carried out in the hotter months of September to November it became apparent that the burrows, being dark and so much cooler than ambient during daylight hours, fulfil all the requirements of a refuge and were clearly used as such on days when temperatures exceeded about 32^°^C.

The dual function of the burrows complicates the analysis of the data because it becomes necessary to make a judgement about which (female) flies are using the sites as a refuge, and which as a larviposition site. Females that are known to have deposited a larva after entering the burrow are clearly in the perinatal group. The classification is more difficult when the female has a third instar larva *in utero*: we tried to reduce the probability of misclassifying flies into the perinatal group by setting a high threshold (estimated proportion of pregnancy completed > 95%) for the inclusion into that category. Flies at this late/full-term stage of pregnancy could reasonably be assumed to be due to produce a larva on the day of capture. While we cannot exclude the possibility that some flies may have been misclassified, support for our classification algorithm comes from the fact that the presumptive refuge and larviposition groups show such distinctly different patterns in terms of changes in catch with calendar date and temperature, and daily time of entry into the burrows.

### Burrows as refuges: Refuges as larviposition sites?

While artificial burrows, designed as larviposition sites, may double as artificial refuges, the reverse does not seem to be true. When artificial refuges were provided with a floor of river sand, fewer than one puparium per week were found in these sites [12]. This emphasises the rather precise conditions that female tsetse appear to require of larviposition sites. When tsetse had the choice of larvipositing in trays filled with either plain sand, or sand covered with leaf litter, 97% of all tsetse puparia were found in the latter site [13]: in that study, as in the present one, it was also found that the vast majority of larvae were deposited in the trays furthest to the back of the burrows. The absence of added leaf litter in Vale’s (1971) refuge flooring, the slightly warmer conditions in the artificial refuges, and the more exposed situation compared with our burrows, will all presumably have reduced the probability that his refuges would be used as larviposition sites [12].

### Species ratios among tsetse adults and puparia sampled from artificial burrows

The greater proportion of *G*. *m*. *morsitans* among pupae collected, as against the proportion among perinatal flies captured (cf Tables 2 and 4) might simply reflect differential proportions of adults of the two species around sites 6 to 9, where puparia were collected, and around sites 1 to 5, where adult flies were trapped. Given, however, that the sites were all within a few hundred metres of each other, and were all in similar habitat, this explanation seems unlikely.

Alternatively the anomaly could reflect a higher level of discrimination among *G*. *pallidipes* in the selection of larviposition sites. In this scenario, among full-term pregnant females entering a burrow, a greater proportion of the *G*. *pallidipes* leave the burrow without larvipositing, and a greater proportion of *G*. *m*. *morsitans* deposit a larva. When there is a trap in place, however, full-term pregnant flies that have entered the burrow are prevented from leaving. Those full-term pregnant female *G*. *pallidipes* that would have left the burrow are now trapped and this changes the balance of the number of perinatal females trapped in favour of this species relative to *G*. *m*. *morsitans*.

Several pieces of evidence are consistent with the above scenario. Firstly, the selection of the trays deepest in the burrows was stronger in *G*. *pallidipes* than in *G*. *m*. *morsitans* (83% vs 74%, Table 1). Perhaps even deeper/longer burrows will elicit improved larviposition by both species, but particularly by *G*. *pallidipes*. Secondly, for *G*. *pallidipes*, the yield per site per day of perinatal females (3.29, Table 5) was 2.7 times higher than the yield of puparia (1.23, Table 1) from burrows where there was no trap and where flies were free to come and go. For *G*. *m*. *morsitans* the yields were very similar, 1.74 and 1.65, respectively. This is consistent with a larger proportion of full-term pregnancy *G*. *pallidipes* entering burrows but then leaving, without larvipositing, if they were allowed to do so. Indeed the above figures suggest that for every ten full-term pregnant female *G*. *pallidipes* entering a burrow, on average perhaps only four would deposit their larvae there. Thirdly, the fact that markedly more *G*. *m*. *morsitans* than *G*. *pallidipes* larvae were deposited in the burrows, in an area where the latter species is clearly more numerous [18], suggests that the artificial burrows were less attractive as larviposition sites to *G*. *pallidipes* than to *G*. *m*. *morsitans*. Fourthly, as reported previously [13], the proportion of *G*. *pallidipes* from natural warthog burrows was double the proportion in artificial burrows.

More carefully controlled experiments will be required to test the tentative idea that many heavily pregnant females entered the burrows but left without larvipositing and that this effect is more accentuated in *G*. *pallidipes*. This suggests an important determinant of larviposition site selection, but the stimuli and responses underlying this selection are unclear. Similarly, it is not clear what underlies the difference in larviposition responses of *G*. *m*. *morsitans* and *G*. *pallidipes*. An impressive array of differences in behavioural responses between tsetse species has been attributed to differences in habitat geometry [19]. Within habitats, differences in behaviour between species, and between males and females of the same species, were attributed to differences in fly size and mobility. It is possible that the differences in larviposition behaviour observed here between the two species has similar causes. *G*. *pallidipes* is a larger and more mobile fly than *G*. *m*. *morsitans* and may, therefore, be able to visit a greater number of potential sites before larvipositing. The study system described here provides ways of addressing this question and other issues.

### Time course of catches of pre-FT and perinatal female tsetse in burrows

Temperature-adjusted catches of males and of pre-FT females continue to increase until mid-October, consistent with the idea that the decline prior to that time, in pupal deposition rates in burrows, reflects a falling probability of tsetse larvipositing in burrows, rather than a decline in the population of full-term-pregnant flies in the area. What is less clear is whether females are reacting to any meteorological or other cues in moving away from using the burrows as sites for larviposition. Regression analysis suggests that there is no effect of either ambient relative humidity or saturation deficit: moreover, the small effect of ambient temperature has a positive coefficient which would tend to increase, rather than decrease, the numbers of larvae deposited in the burrows as the hot season progresses. We cannot exclude the possibility that flies were reacting to changes in humidity within burrows, which was not measured here, but should be monitored in future studies.

Reduced use of warthog burrows as larviposition sites during October and November, reflected both in the reduction in the absolute numbers of perinatal flies captured in burrows, and of puparia found in these sites, may be adaptive. When heavy rains fall, the burrows become waterlogged and may even flood, and any pupae present would presumably perish. In 1998 the first rains only fell on 19 November, whereas the decline in captures of perinatal flies started a month before this time. Nonetheless, it is not uncommon for heavy rain to fall in the first half of November; in 5/10 years in the period 1989–1998 > 12.5 mm of rain were recorded at Rekomitjie by 15 November. Larvae deposited in the last half of October might not, therefore, have emerged by the time the first heavy rains fell. Over the same period 0/10 years had recorded this level of rain by the end of October. There is therefore little danger of flooding for any flies deposited in burrows during September, but this danger increases in October and particularly November.

### Age structures

It has been argued that refuges provide the least biased sample of tsetse currently available—at least in terms of age structure and pregnancy stage [20,21]. The similarity between the age structures of perinatal females and those apparently using the burrows as a refuge is then consistent with the idea that all ages of fly use the burrows as larviposition sites with approximately the same probability.

### Limitations, conclusions

For both species, perinatal flies were most often caught before the hottest time of the day (at about 1500h): 45–65% were captured by 1230h on any given day and most often larviposited in the collection tube shortly thereafter. We do not know, however, when the flies would have larviposited if they had not been caught, nor even whether they would have larviposited in the burrow where they were caught had they not been trapped. Further field studies are required to better estimate the timing of larviposition in the field.

Interpretation of catches from burrow traps is also complicated by the unknown efficiency of the traps. On one hand the presence of the trap may have discouraged some flies from entering the burrow. Conversely, traps occasionally contained a pupa that could not have been produced by any of the flies in the trap—implying that a postpartum fly had escaped. A better idea of the number of flies entering the burrows could be obtained by deploying electric nets [22] around the entrance of the burrow and/or even inside it.

The estimate that 60% of full-term *G*. *pallidipes* leave a burrow that they have entered, without larvipositing there, suggests that we should seek ways of improving the burrows such that females are more likely to larviposit in the burrow they first enter. This study, and an earlier one [13], made no progress in improving on the original burrow design. The use of the burrows as larviposition sites also seems to be limited to about four months of the year and our knowledge of the behaviour of perinatal flies in the field is thus currently limited to just two species of tsetse during a single season. At other times of the year pupae are deposited along the edges of dried up water-courses, in rot holes in trees and under fallen logs. Artificial versions of the latter two sites might be used to sample larvipositing flies at other times of the year at Rekomitjie, and indeed to sample other species in other parts of Africa.

Currently, the burrows capture system provides the only method for collecting perinatal tsetse in the field, providing thereby a unique opportunity to study the physiology of female tsetse and the larvae that they have just deposited. Future papers will address this interesting new area of tsetse field biology.

## References

[1] Phelps RJ, Jackson PJ (1971) Factors influencing the moment of larviposition and eclosion in *Glossina morsitans orientalis* Vanderplank (Diptera: Muscidae). Journal of the Entomological Society of southern Africa 34: 145–157.

[2] Nash TAM, Trewern MA (1972) Hourly distribution of larviposition by Glossina austeni Newst. and *G*. *morsitans morsitans* Westw. (Dipt., Glossinidae). Bulletin of Entomological Research 61: 693–700.

[3] Rowcliffe C, Finlayson LH (1982) Active and resting behaviour of virgin and pregnant females of *Glossina morsitans morsitans* Westwood (Diptera: Glossinidae) in the laboratory. Bulletin of Entomological Research 72: 271–288.

[4] Brady J, Gibson G (1983) Activity patterns in pregnant tsetse flies, *Glossina morsitans* . Physiological Entomology 8: 359–369.

[5] Abdel Karim EI, Brady J (1984) Changing visual responsiveness in pregnant and larvipositing tsetse flies, *Glossina morsitans* . Physiological Entomology 9: 125–131.

[6] Leak S (1998) Tsetse biology and ecology: their role in the epidemiology and control of trypanosomosis: CABI Publishing 592 p.

[7] Robinson MW, Baker PS, Finlayson LH (1985) Influence of temperature changes on larviposition rhythm in the tsetse fly, *Glossina morsitans* . Physiological Entomology 10: 215–220.

[8] Tobe SS, Langley PA (1978) Reproductive physiology of *Glossina* . Annual Review of Entomology 23: 283–307. 343707 10.1146/annurev.en.23.010178.001435

[9] Randolph SE, Rogers DJ, Dransfield RD, Brightwell R (1991) Trap catches, nutritional condition and the timing of activity of the tsetse fly *Glossina longipennis* (Diptera, Glossinidae). Bulletin of Entomological Research 81: 455–464.

[10] Phelps RJ, Simmonds AM, Parsons R (1966) Pupal collection and respiratory physiology. Rhodesia Agricultural Research Council Annual Report 1966 Salisbury, Rhodesia: 77–79.

[11] Phelps RJ, Burrows PM (1969) Prediction of the puparial duration in *Glossina morsitans orientalis* Vanderplank under field conditions. Journal of Applied Ecology 57: 227–257.

[12] Vale GA (1971) Artificial refuges for tsetse flies (*Glossina* spp). Bulletin of Entomological Research 61: 331–350.

[13] Muzari MO, Hargrove JW (2005) Artificial larviposition sites for field collections of the puparia of tsetse flies *Glossina pallidipes* and *G*. *m*. *morsitans* (Diptera: Glossinidae). Bulletin of Entomological Research 95: 221–229. 15960876 10.1079/ber2004354

[14] Torr SJ, Hargrove JW (1999) Behaviour of tsetse (Diptera: Glossinidae) during the hot season in Zimbabwe: the interaction of micro-climate and reproductive status. Bulletin of Entomological Research 89: 365–379.

[15] Challier A (1965) Amélioration de la méthode de détermination de l'âge physiologique des glossines. Études faites sur Glossina palpalis palpalis Vanderplank, 1949. Bulletin de la Société de Pathologie Exotique 58: 250–259. 5898576

[16] Hargrove JW (1994) Reproductive rates of tsetse flies in the field in Zimbabwe. Physiological Entomology 19: 307–318.

[17] Hargrove JW (1995) Towards a general rule for estimating the stage of pregnancy in field-caught tsetse flies. Physiological Entomology 20: 213–223.

[18] Hargrove JW (1981) Discrepancies between estimates of tsetse fly populations using mark-recapture and removal trapping techniques. Journal of Applied Ecology 18: 737–748.

[19] Vale GA HJ, Solano P, Courtin F, Rayaisse J-B, Lehane MJ, Esterhuizen J, Naki Tirados N, Torr SJ (2014) Explaining the host-finding behavior of blood-sucking insects: computerized simulation of the effects of habitat geometry on tsetse fly movement. PLoS NTD 8. 10.1371/journal.pntd.0002901 PMC4055578 24921243

[20] Hargrove JW (1999) Lifetime changes in the nutritional characteristics of female tsetse *Glossina pallidipes* caught in odour-baited traps. Medical and Veterinary Entomology 13: 165–176. 10484162 10.1046/j.1365-2915.1999.00153.x

[21] Hargrove JW (1999) Nutritional levels of female tsetse *Glossina pallidipes* from artificial refuges. Medical and Veterinary Entomology 13: 150–164. 10484161 10.1046/j.1365-2915.1999.00152.x

[22] Vale GA (1974) New field methods for studying responses of tsetse flies (Diptera, Glossinidae) to hosts. Bulletin of Entomological Research 64: 199–208.

